# Efficient whole-cell oxidation of α,β-unsaturated alcohols to α,β-unsaturated aldehydes through the cascade biocatalysis of alcohol dehydrogenase, NADPH oxidase and hemoglobin

**DOI:** 10.1186/s12934-021-01511-8

**Published:** 2021-01-19

**Authors:** Yan Qiao, Can Wang, Yin Zeng, Tairan Wang, Jingjing Qiao, Chenze Lu, Zhao Wang, Xiangxian Ying

**Affiliations:** 1 Key Laboratory of Bioorganic Synthesis of Zhejiang Province, College of Biotechnology and Bioengineering, Zhejiang University of Technology, Hangzhou, 310014 China; 2 College of Life Sciences, China Jiliang University, Hangzhou, 310018 China

**Keywords:** α,β-Unsaturated aldehydes, Alcohol dehydrogenase, NADPH oxidase, NADP^+^ regeneration, Enzyme fusion

## Abstract

**Background:**

α,β-Unsaturated aldehydes are widely used in the organic synthesis of fine chemicals for application in products such as flavoring agents, fragrances and pharmaceuticals. In the selective oxidation of α,β-unsaturated alcohols to the corresponding α,β-unsaturated aldehydes, it remains challenging to overcome poor selectivity, overoxidation and a low atom efficiency in chemical routes.

**Results:**

An *E. coli* strain coexpressing the NADP^+^-specific alcohol dehydrogenase YsADH and the oxygen-dependent NADPH oxidase TkNOX was constructed; these components enabled the NADP^+^ regeneration and catalyzed the oxidation of 100 mM 3-methyl-2-buten-1-ol to 3-methyl-2-butenal with a yield of 21.3%. The oxygen supply was strengthened by introducing the hemoglobin protein VsHGB into recombinant *E. coli* cells and replacing the atmosphere of the reactor with pure oxygen, which increased the yield to 51.3%. To further improve catalytic performance, the *E. coli* cells expressing the multifunctional fusion enzyme YsADH-(GSG)-TkNOX-(GSG)-VsHGB were generated, which completely converted 250 mM 3-methyl-2-buten-1-ol to 3-methyl-2-butenal after 8 h of whole-cell oxidation. The reaction conditions for the cascade biocatalysis were optimized, in which supplementation with 0.2 mM FAD and 0.4 mM NADP^+^ was essential for maintaining high catalytic activity. Finally, the established whole-cell system could serve as a platform for the synthesis of valuable α,β-unsaturated aldehydes through the selective oxidation of various α,β-unsaturated alcohols.

**Conclusions:**

The construction of a strain expressing the fusion enzyme YsADH-(GSG)-TkNOX-(GSG)-VsHGB achieved efficient NADP^+^ regeneration and the selective oxidation of various α,β-unsaturated alcohols to the corresponding α,β-unsaturated aldehydes. Among the available redox enzymes, the fusion enzyme YsADH-(GSG)-TkNOX-(GSG)-VsHGB has become the most recent successful example to improve catalytic performance in comparison with its separate components.

## Background

α,β-Unsaturated aldehydes serve as important intermediates in the organic synthesis of an extended range of fine chemicals for use in products such as flavoring agents, fragrances and pharmaceuticals [1–3]. For example, citral (3,7-dimethyl-2,6-octadien-1-al) is in high demand for the production of ionones, vitamins A and E, and carotenoids [4]. α,β-Unsaturated aldehydes can be obtained through the selective oxidation of the corresponding α,β-unsaturated alcohols via either chemical catalysis or biocatalysis [3, 5–7]. Traditional chemical oxidation methods have required the use of equimolar amounts of oxidizing reagents, whose atom efficiency is relatively low. Moreover, the practical application of chemical routes is often limited by overoxidation, poor selectivity and the use of organic solvents and toxic compounds [8]. Alternatively, biocatalytic oxidation has raised great interest because of its excellent selectivity and environmentally friendly nature as well as the mild reaction conditions involved [9–11].

Both alcohol oxidases and alcohol dehydrogenases are attractive biocatalysts used for the selective oxidation of α,β-unsaturated alcohols. Alcohol oxidases utilize the cheap mild oxidant molecular oxygen and generally form highly reactive hydrogen peroxide as a byproduct [9]. In most cases, the toxicity of hydrogen peroxide can be partially avoided by the catalytic dismutation of H_2_O_2_ into O_2_ and water using catalase. A prominent example is the recombinant aryl alcohol oxidase from *Pleurotus eryngii* used for the selective oxidation of *trans*-2-hexen-1-ol to *trans*-2-hexenal, in which the turnover number exceeds 2.2 million [3]. The enzyme in its active form is obtained from inclusion bodies in a cell extract, which prevents the use of whole-cell biotransformation [12]. Compared with isolated enzymes, the use of whole-cell catalysts simplifies the procedure, reduces costs and improves the enzyme stability. Despite great potential, the number of characterized alcohol oxidases is very limited, in contrast to the wide array of alcohol dehydrogenases with various substrate specificities [7, 13–16]. Alcohol dehydrogenases catalyze reversible oxidation reactions and require NAD(P)^+^, suggesting that efficient NAD(P)^+^ regeneration is needed to shift the reaction equilibrium toward product formation [11, 17].

NAD(P)^+^ regeneration can be substrate-coupled or enzyme-coupled, where the latter does not require the use of excess cosubstrate to ensure the efficient oxidation [11]. Among enzyme-coupled approaches, the cascade biotransformation of alcohol dehydrogenase and NAD(P)H oxidase is much more atomically efficient for NAD(P)^+^ recycling, where the direct oxidation of NAD(P)H by molecular oxygen forms hydrogen peroxide or water [18]. In particular, water-forming NAD(P)H oxidase is ideal because there are no byproducts [19, 20]. In multiple enzymatic cascade reactions, the possibilities and advantages of enzyme fusions have been explored for various enzyme types, including fusions of redox enzymes [21–26]. Through this approach, enzymes can be produced simultaneously and are colocalized in cells. The fusion of ADH with NOX to perform alcohol oxidation supported the rapid regeneration of NADP^+^, and the cascade reaction was more efficient than the separate enzymes [27].

**Scheme 1:**
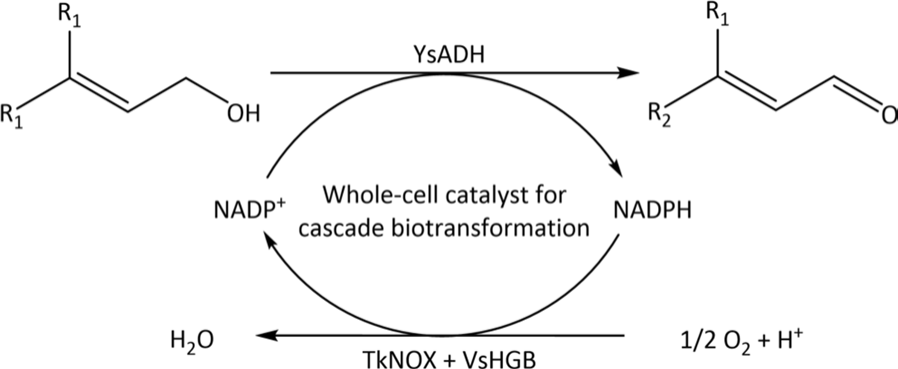
Selective oxidation of α,β-unsaturated alcohols to α,β-unsaturated aldehydes using *E. coli* cells coexpressing YsADH alcohol dehydrogenase, TkNOX NADPH oxidase and VsHGB hemoglobin

Our previous work showed that NADP^+^-specific YsADH, an α,β-unsaturated alcohol dehydrogenase from *Yokenella* sp. WZY002, exhibited high activity and stability in the selective oxidation of crotonyl alcohol to crotyonyl aldehyde [7]. In this work, the selective oxidation of 3-methyl-2-buten-1-ol to 3-methyl-2-butenal was chosen as the model reaction, considering that both 3-methyl-2-buten-1-ol and 3-methyl-2-butenal serve as important organic synthesis intermediates. The use of the NADPH oxidase TkNOX together with VsHGB hemoglobin, capable of binding and releasing oxygen, was explored for the regeneration of NADP^+^ and the improvement of catalytic efficiency of selective oxidation [28–30] (Scheme 1). Moreover, the fusion protein of YsADH-(linker)-TkNOX-(linker)-VsHGB was constructed to increase the catalytic performance, resulting in the efficient whole-cell oxidation of various α,β-unsaturated alcohols to the corresponding α,β-unsaturated aldehydes.

## Results and discussion

### Cascade catalysis of alcohol dehydrogenase, NADPH oxidase and hemoglobin

**Fig. 1:**
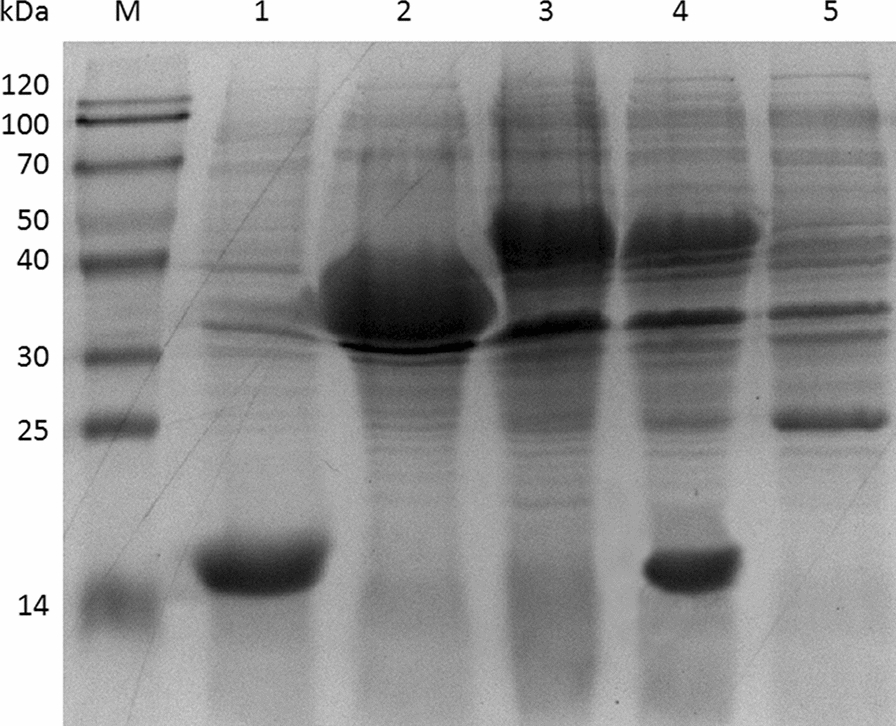
SDS-PAGE analysis of YsADH, TkNOX and/or VsHGB in the cell-free extracts. Lane M, marker; lane 1, expression of VsHGB (15.5 kDa) alone; lane 2, expression of YsADH (33.2 kDa) alone; lane 3, coexpression of YsADH (33.2 kDa) and TkNOX (43.4 kDa); lane 4, coexpression of YsADH (33.2 kDa), TkNOX (43.4 kDa) and VsHGB (15.5 kDa); lane 5, no induction of cells coexpressing YsADH, TkNOX or VsHGB as the control. The percentage of acrylamide in the resolving gel was 12%. The value in the bracket represents the apparent molecular mass of YsADH, TkNOX or VsHGB

Alcohol dehydrogenase from *Yokenella* sp. WZY002 is highly active in the oxidation of various α,β-unsaturated alcohols to the corresponding α,β-unsaturated aldehydes [7]. To be compatible with YsADH activity (e.g., temperature and pH optima), a thermostable NADPH oxidase from *Thermococcus kodakaraensis* was chosen to catalyze the oxidation of NADPH, predominantly converting O_2_ to H_2_O [29]. A recombinant strain expressing YsADH alone and a strain coexpressing YsADH and TkNOX were successfully constructed and induced, and the formation of inclusion bodies was not observed in these strains (Fig. 1, Additional file 1: Figure S1). It was noted that the cooccurrence of YsADH and TkNOX significantly affected the expression level of YsADH. In particular, the activity of YsADH was reduced from 3568 U/g (expression of YsADH alone) to 650 U/g (coexpression of YsADH and TkNOX) (Additional file 1: Table S1). The whole-cell catalyst expressing YsADH alone catalyzed the oxidation of 100 mM 3-methyl-2-buten-1-ol to 3-methyl-2-butenal with a yield of 11.75% (Table 1). The yield of 3-methyl-2-butenal was increased up to 21.3% when cells coexpressing YsADH and TkNOX were used as biocatalysts, indicating that alcohol oxidation benefited from the NADP^+^ regeneration.

**Table 1:** Selective oxidation of 3-methyl-2-buten-1-ol to 3-methyl-2-butenal catalyzed by cells expressing YsADH, TkNOX and/or VsHGB

The protein(s) expressed in *E. coli* cells	Yield of 3-methyl-2-butenal (%)^a^
YsADH	11.75 ± 0.22
YsADH and TkNOX	21.3 ± 0.79
YsADH, TkNOX and VsHGB	35.48 ± 1.47
YsADH, TkNOX and VsHGB^b^	51.3 ± 2.03

The reaction mixture (5 ml) contained 100 mM 3-methyl-2-buten-1-ol, 0.3 g lyophilized cells, 0.4 mM NADP^+^, 0.2 mM FAD, and 50 mM Tris-HCl (pH 8.0). The reaction was carried out at 45 °C and 600 rpm for 2 h. The values are presented as the standard deviation from triplicate measurements

^a^The yield of 3-methyl-2-butenal was calculated with the following equation: yield (%) = product formed (mM)/(product formed (mM) + substrate remaining (mM)) × 100%

^b^The atmosphere of the reactor was replaced with pure oxygen

Whole-cell oxidation catalyzed by oxygen-dependent enzymes might be restricted by the oxygen supply because of the limited solubility of oxygen in water [30]. To improve the supply of oxygen, the gene encoding hemoglobin from *Vitreoscilla stercoraria* was further introduced into the recombinant cells coexpressing YsADH and TkNOX, giving rise to a strain coexpressing YsADH, TkNOX and VsHGB [28]. Cell-free extracts of the cells coexpressing YsADH, TkNOX and VsHGB were prepared, and the activities of YsADH and TkNOX were determined to be 613 U/g and 1542 U/g, respectively. The use of the strain coexpressing YsADH, TkNOX and VsHGB increased the yield of 3-methyl-2-butenal to 35.48%. In addition, the atmosphere of the reactor was replaced with pure oxygen to increase the supply of oxygen, in which the yield of 3-methyl-2-butenal reached 51.3% (Table 1). To determine whether overoxidation of the product occurred, the possible side products isovaleraldehyde, isovaleric acid and 3,3-dimethylacrylic acid were verified by gas chromatography. The results indicated that none of these products were detectable, demonstrating that the reaction conditions were mild enough for the oxidation of 3-methyl-2-buten-1-ol to 3-methyl-2-butenal.

### Construction of YsADH/TkNOX/VsHGB fusion proteins and their catalytic performance

**Fig. 2:**
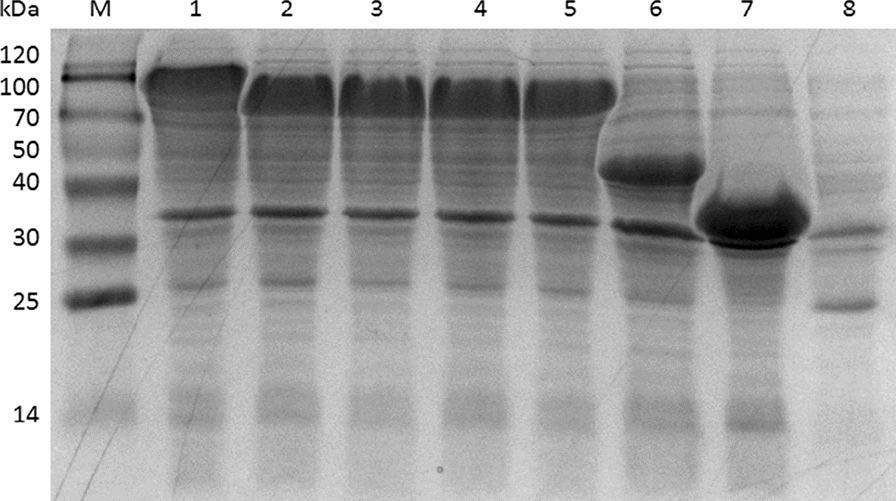
SDS-PAGE analysis of the YsADH-(linker)-TkNOX and YsADH-(linker)-TkNOX-(linker)-VsHGB fusion proteins in cell-free extracts. Lane M, marker; lane 1, expression of YsADH-(GSG)-TkNOX-(GSG)-VsHGB (99.7 kDa); lane 2, expression of YsADH-(GSG)-TkNOX (80.7 kDa); lane 3, expression of YsADH-(GSG)_2_-TkNOX (80.7 kDa); lane 4, expression of YsADH-(GGGGS)-TkNOX (80.7 kDa); lane 5, expression of YsADH-(GGGGS)_2_-TkNOX (80.7 kDa); lane 6, expression of TkNOX (42.7 kDa); lane 7, expression of YsADH (32.7 kDa); lane 8, uninduced cells coexpressing YsADH, TkNOX and VsHGB as the control. The percentage of acrylamide in the resolving gel was 12%. The value in the bracket represents the apparent molecular mass of the fusion enzyme, YsADH or TkNOX

**Fig. 3:**
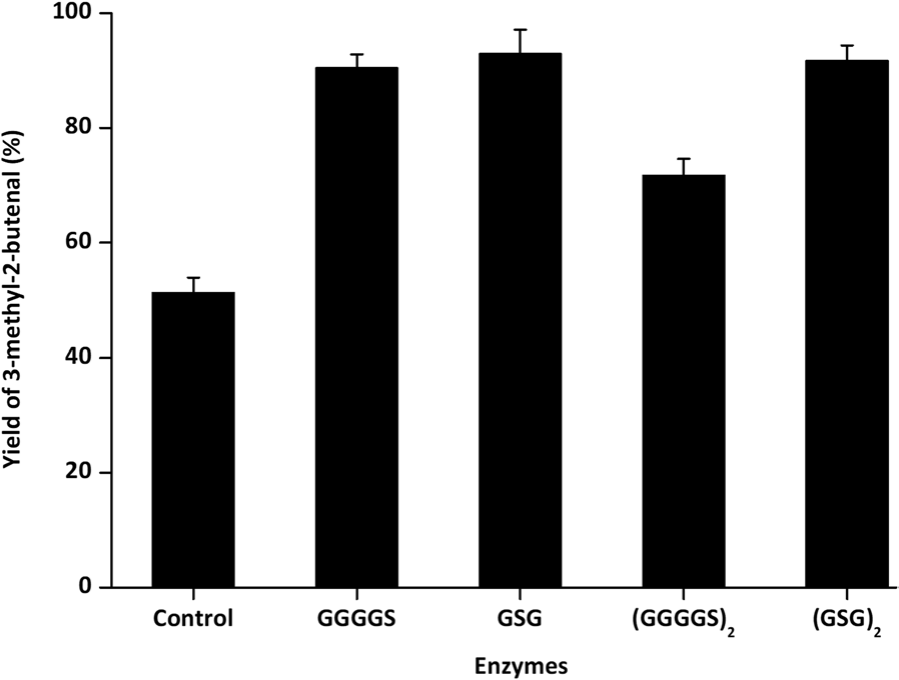
Effect of the linker type on the catalytic performance of the fusion enzyme YsADH-(linker)-TkNOX. The control represents the *E. coli* cells coexpressing YsADH, TkNOX and VsHGB separately. The reaction mixture (5 ml) contained 100 mM 3-methyl-2-buten-1-ol, 0.3 g lyophilized cells, 0.4 mM NADP^+^, 0.2 mM FAD, and 50 mM Tris-HCl (pH 8.0). The reaction was carried out at 45 °C and 600 rpm for 2 h. Standard deviations are indicated in the diagram (n = 3)

In redox reactions, enzyme fusions have been approved as an efficient approach to support the rapid regeneration of NADP^+^ [27]. To test whether the fusion of YsADH and TkNOX could improve the selective oxidation of 3-methyl-2-buten-1-ol, a YsADH-TkNOX fusion with a GGGGS linker was initially constructed and successfully induced (Fig. 2). Whole-cell catalysis in cells expressing YsADH-(GGGGS)-TkNOX indicated that the yield of 3-methyl-2-butenal was significantly increased from 51.3% to 80.57% (Fig. 3). It was suggested that the length of the flexible linkers might affect the activity of the fusion proteins [31]. Then, fusion genes were constructed and compared by linking the YsADH and TkNOX with GGGGS as well as other peptide linkers ((GGGGS)_2_, GSG and (GSG)_2_). All four resulting fusion genes were transformed into *E. coli* and then induced, and the formation of inclusion bodies was not observed in these strains (Fig. 2, Additional file 1: Figure S2). In contrast to the strain coexpressing the individual enzymes, all of the fusion proteins exhibited much higher catalytic efficiency (Fig. 3). The fusion enzymes with the GSG, (GSG)_2_ or GGGGS linker showed similar activity against the substrate 3-methyl-2-buten-1-ol, which was higher than that of the enzyme with the (GGGGS)_2_ linker. The GSG linker was finally used to generate cells expressing the fusion protein YsADH-(GSG)-TkNOX-(GSG)-VsHGB or VsHGB-(GSG)-TkNOX-(GSG)-YsADH (Additional file 1: Figure S3), and both of these fusions completely converted 100 mM 3-methyl-2-buten-1-ol to 3-methyl-2-butenal after 2 h of whole-cell oxidation. The functional expression of both the YsADH and TkNOX enzymes was also verified by measuring their activities in cell-free extracts comprising YsADH-(GSG)-TkNOX-(GSG)-VsHGB or VsHGB-(GSG)-TkNOX-(GSG)-YsADH. The specific activities of YsADH and TkNOX in the cell-free extracts comprising YsADH-(GSG)-TkNOX-(GSG)-VsHGB were determined to be 801 U/g and 983 U/g, respectively, while those in the cell-free extract comprising VsHGB-(GSG)-TkNOX-(GSG)-YsADH were 752 U/g and 1064 U/g, respectively (Additional file 1: Table S1). In the previous case involving the fusions of alcohol dehydrogenase with cyclohexanone monooxygenase, the fusion protein with the order alcohol dehydrogenase-cyclohexanone monooxygenase showed low to no alcohol dehydrogenase activity [25]. The fusion proteins in which the components were ordered YsADH-(GSG)-TkNOX-(GSG)-VsHGB and VsHGB-(GSG)-TkNOX-(GSG)-YsADH not only maintained high levels of both YsADH and TkNOX activities, but the ratio of YsADH and TkNOX activities was also more optimal than that in the cells coexpressing YsADH, TkNOX and VsHGB separately.

### Evaluation of factors affecting the catalytic efficiency of whole-cell catalysts expressing YsADH-(GSG)-TkNOX-(GSG)-VsHGB

**Fig. 4:**
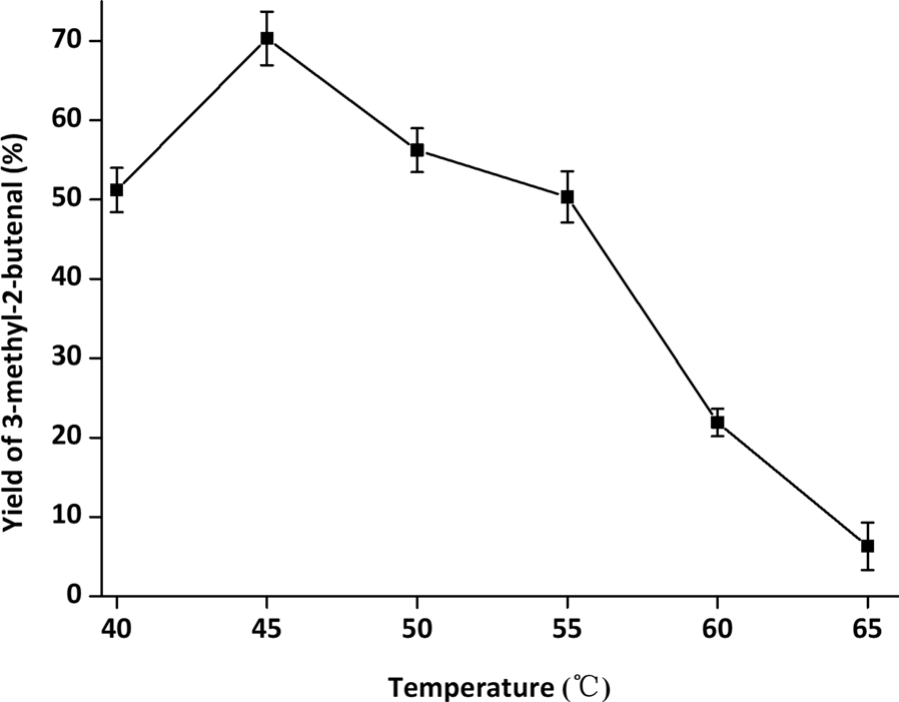
Effect of temperature on the catalytic performance of cells expressing the fusion enzyme YsADH-(GSG)-TkNOX-(GSG)-VsHGB. The reaction mixture (5 ml) contained 100 mM 3-methyl-2-buten-1-ol, 0.1 g lyophilized cells, 0.4 mM NADP^+^, 0.2 mM FAD, and 50 mM Tris-HCl (pH 8.0). The reactions were carried out at 40–65 °C and 600 rpm for 2 h. Standard deviations are indicated in the diagram (n = 3)

**Fig. 5:**
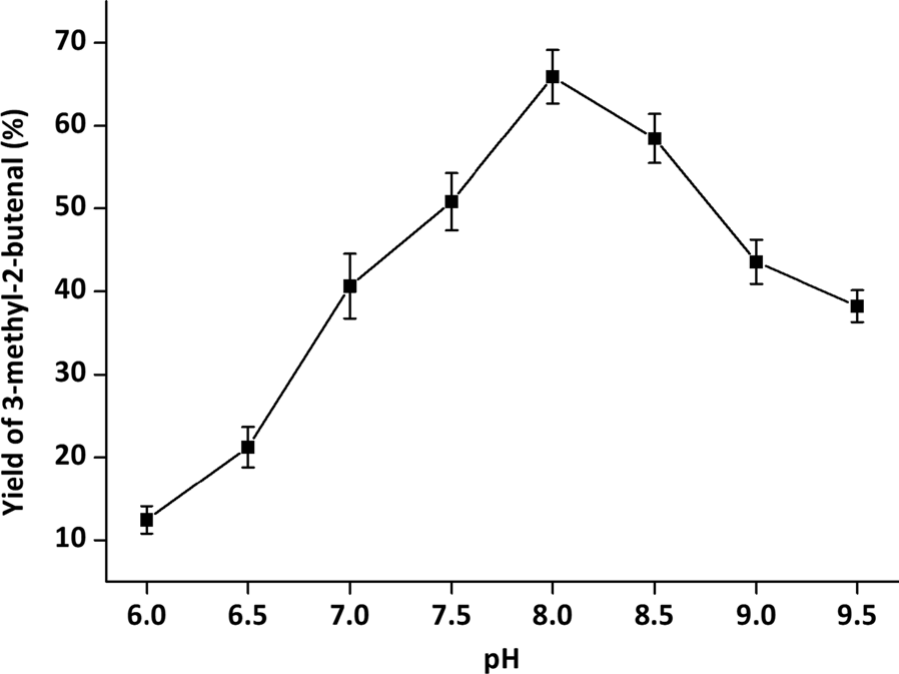
Effect of pH on the catalytic performance of cells expressing the fusion enzyme YsADH-(GSG)-TkNOX-(GSG)-VsHGB. The reaction mixture (5 ml) contained 100 mM 3-methyl-2-buten-1-ol, 0.1 g lyophilized cells, 0.4 mM NADP^+^, 0.2 mM FAD, 50 mM buffer (PIPES, pH 6.0, 6.5 or 7.0; Tris-HCl, pH 7.5, 8.0, 8.5 or 9.0; CAPS, pH 9.5). The reactions were carried out at 45 °C and 600 rpm for 2 h. Standard deviations are indicated in the diagram (n = 3)

**Fig. 6:**
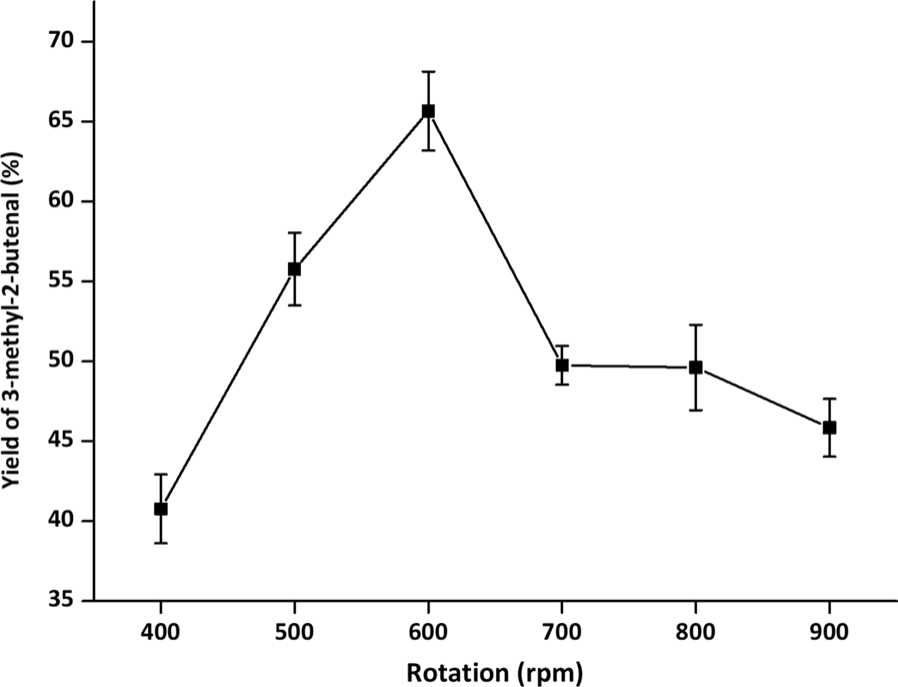
Effect of rotation on the catalytic performance of cells expressing the fusion enzyme YsADH-(GSG)-TkNOX-(GSG)-VsHGB. The reaction mixture (5 ml) contained 100 mM 3-methyl-2-buten-1-ol, 0.1 g lyophilized cells, 0.4 mM NADP^+^, 0.2 mM FAD, and 50 mM Tris-HCl (pH 8.0). The reactions were carried out at 45 °C and 400–900 rpm for 2 h. Standard deviations are indicated in the diagram (n = 3)

**Fig. 7:**
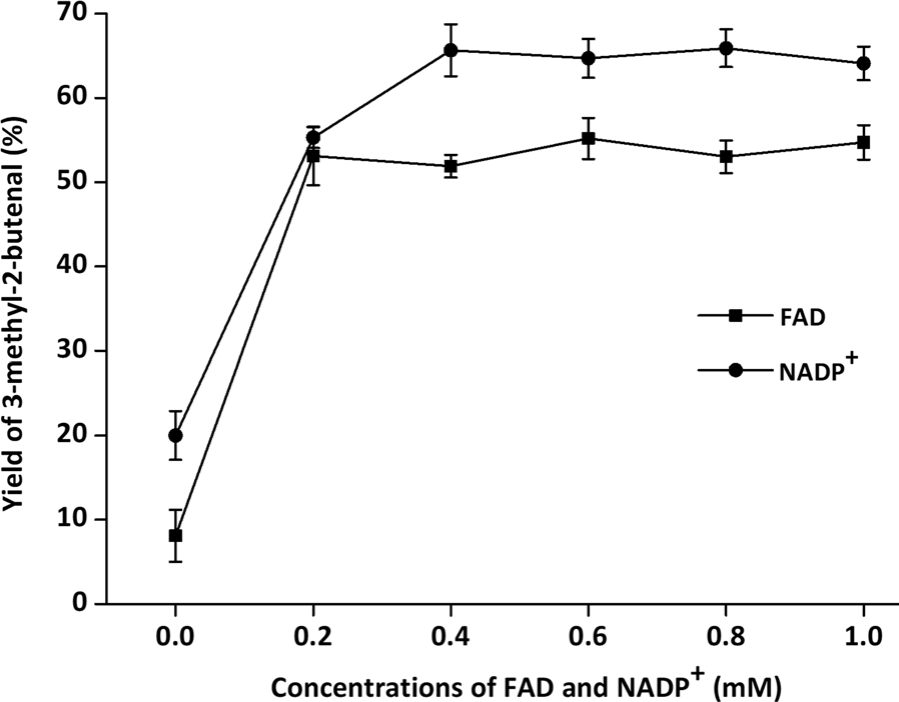
Effect of FAD and NADP^+^ coenzymes on the catalytic performance of cells expressing the YsADH-(GSG)-TkNOX-(GSG)-VsHGB fusion enzyme. The reaction mixture (5 ml) contained 100 mM 3-methyl-2-buten-1-ol, 0.1 g lyophilized cells, 0–1.0 mM NADP^+^, 0–1.0 mM FAD, and 50 mM Tris-HCl (pH 8.0). The effect of the FAD concentration was investigated at an NADP^+^ concentration of 0.2 mM, and that effect of the NADP^+^ concentration was then evaluated at an FAD concentration of 0.2 mM. All the reactions were carried out at 45 °C and 600 rpm for 2 h. Standard deviations are indicated in the diagram (n = 3)

**Fig. 8:**
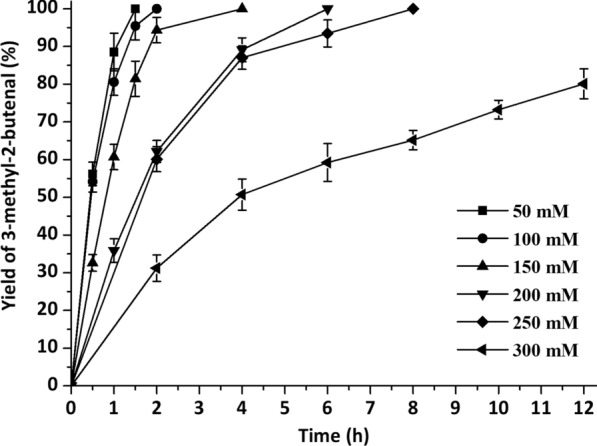
Time courses of alcohol oxidation at higher substrate concentrations. The reaction mixture (5 ml) contained 50–300 mM 3-methyl-2-buten-1-ol, 0.3 g lyophilized cells, 0.4 mM NADP^+^, 0.2 mM FAD, and 50 mM Tris-HCl (pH 8.0). The reaction was carried out at 45 °C and 600 rpm for 2 h. Standard deviations are indicated in the diagram (n = 3)

During whole-cell cascade biocatalysis, multiple reactions are run in the same pot, and each reaction often does not reach the optimal conditions [32]. To achieve optimal catalytic efficiency, it is essential to orchestrate the catalytic performance of YsADH, TkNOX and VsHGB in the YsADH-(GSG)-TkNOX-(GSG)-VsHGB fusion protein. Hence, various factors, such as temperature, pH, rotation, FAD and NADP^+^ concentrations and the substrate concentration, were investigated when the cells comprising YsADH-(GSG)-TkNOX-(GSG)-VsHGB were chosen as whole-cell catalysts. To magnify the differences in catalytic performances, the biocatalyst loading was reduced to 0.1 g lyophilized cells in a 5 ml reaction mixture. The influence of the reaction temperature was determined over a range of 40–65 °C, and the highest yield of 3-methyl-2-butenal (70.35%) was observed at 45 °C (Fig. 4). When the temperature was greater than 45 °C, the product yield decreased as the temperature rose. Since TkNOX is highly thermostable and thermoactive, the thermal optimum might mainly correspond to that of YsADH [7, 29]. To determine the optimal pH, the reaction was carried out at pH levels ranging from 6.0 to 9.5 at 45 °C. The highest production was detected at pH 8.0 (Fig. 5), which was consistent with the pH optima of both YsADH and TkNOX [7, 29]. Similarly, rotation was optimized to be 600 rpm (Fig. 6), considering both mass transfer and shear force. It has been suggested that the expression of FAD-dependent NOXs in *E. coli* might result in poor activity due to the absence of FAD [33]. The yield of 3-methyl-2-butenal (53.1%) in the presence of 0.2 mM exogenous FAD was 6.56 times higher than that without the addition of exogenous FAD. When the FAD concentration was set as 0.2 mM, 0.4 mM NADP^+^ was sufficient for maintaining high activity in the oxidation of 3-methyl-2-buten-1-ol (Fig. 7). When the substrate concentration was increased in a stepwise manner, typical time courses under biocatalyst loading of 0.3 g lyophilized cells in a 5 ml reaction mixture are shown in Fig. 8. The times required to completely convert 50, 100, 150, 200 and 250 mM 3-methyl-2-buten-1-ol to 3-methyl-2-butenal were 1.5, 2, 4, 6 and 8 h, respectively. A further increase in the substrate concentration to 300 mM resulted in an 80.1% yield of 3-methyl-2-butenal within 12 h, and the decrease in catalytic efficiency might be attributed to cell disintegration from the accumulation of hydrophobic compounds and/or the enzyme inactivation [12].

### Oxidation of various α,β-unsaturated alcohols to α,β-unsaturated aldehydes

To expand the applicability of the established whole-cell oxidation, the use of various α,β-unsaturated alcohols to produce α,β-unsaturated aldehydes, some of which are of great industrial interest, were examined (Table 2). For example, retinol oxidation was performed to form retinal, which might be further converted to carotene [34]. The oxidation of farnesol to farnesal is a key step in the synthesis of vitamin E using farnesol as starting material [35, 36]. Similar to the case of 3-methyl-2-buten-1-ol, 200 mM crotyl alcohol was completely converted to crotonaldehyde after 6 h of oxidation. The yield of crotonaldehyde (96.70%, 8 h) in the initial presence of 300 mM crotyl alcohol was even greater than that (80.1%, 12 h) in the initial presence of 300 mM 3-methyl-2-buten-1-ol. The oxidation of *trans*-2-hexenol, geraniol and nerol for 8 h resulted in similar catalytic performances (83.35–93.46% yields), whereas that of cinnamyl alcohol resulted in a relatively lower yield of cinnamyl aldehyde (47.49%). The results suggested that the catalytic efficiency might be associated with the solubility and/or molecule size of the substrate. In addition, enzyme inactivation through covalent modification could not be ignored since the carbonyl group of α,β-unsaturated aldehydes (e.g., crotonaldehyde) can form a Schiff base with the lysine side chain, and the cysteine thiol group of the enzyme then attacks the C_β_ atom of the C=C band of α,β-unsaturated aldehydes [37]. Efforts aimed at protein engineering with the goal of mitigating activity inhibition are currently underway in our laboratory.

**Table 2:**
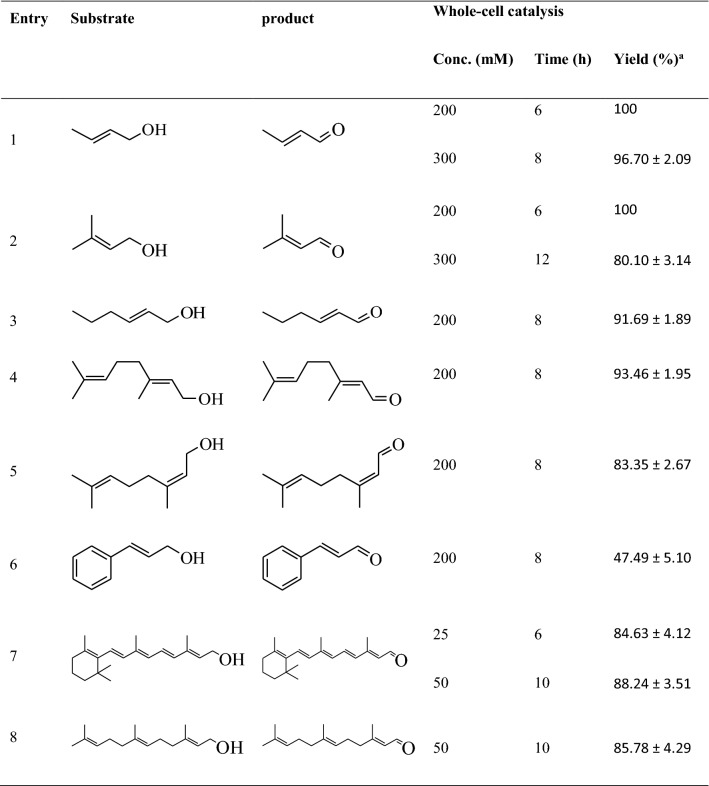
Oxidation of various α,β-unsaturated alcohols under optimized conditions

The reaction mixture (5 ml) contained α,β-unsaturated alcohols, 0.3 g lyophilized cells, 0.4 mM NADP^+^, 0.2 mM FAD, and 50 mM Tris-HCl (pH 8.0). The reaction was carried out at 45 °C and 600 rpm. The values are presented with the standard deviation from triplicate measurements

^a^The yield was calculated with the following equation: yield (%) = product formed (mM)/(product formed (mM) + substrate remaining (mM)) × 100%

## Conclusions

As an alternative to the use of alcohol oxidase and catalase, this study developed a method for the whole-cell oxidation of α,β-unsaturated alcohols to α,β-unsaturated aldehydes based on the combination of YsADH alcohol dehydrogenase and TkNOX NADPH oxidase. Both NADP^+^-dependent YsADH and oxygen-dependent TkNOX were highly active and compatible with temperature and pH optima. The catalytic efficiency and the NADP^+^ regeneration achieved during the whole-cell oxidation were enhanced through the improvement of the oxygen supply, the overexpression of YsADH, TkNOX and VsHGB fusion proteins, and the optimization of reaction conditions. In the case of 3-methyl-2-buten-1-ol oxidation, the whole-cell cascade catalysis system enabled efficient alcohol oxidation but also overcame the poor selectivity and over oxidation that occur frequently in chemical synthesis. The established whole-cell system could be tuned to achieve the synthesis of various α,β-unsaturated aldehydes from the selective oxidation of α,β-unsaturated alcohols.

## Methods

### Chemicals, enzymes, plasmids and strains

The standards α,β-unsaturated alcohols and aldehydes were obtained from Sigma-Aldrich (Shanghai) Trading Co., Ltd. (Shanghai, China). Other chemicals of analytical grade were purchased from Sangon Biotech Co. Ltd (Shanghai, China) or Shanghai Jingchun Reagent Co., Ltd (Shanghai, China). The kits and the enzymes for gene manipulation were obtained from Takara Biomedical Technology Co., Ltd. (Beijing, China). The pET28a and pACYCDuet-1 vectors were used for the over-expression of the enzymes, and the *E. coli* strain BL21(DE3) was used as the host. *E. coli* cultures were grown routinely in LB medium at 37 °C for 12 h.

### Cloning and overexpression of YsADH, TkNOX and VsHGB in *E. coli* cells

The DNA sequences encoding YsADH (GenBank accession number: KF887947), TkNOX (BAD85488) and VsHGB (AAA27584) were codon-optimized and synthesized at Vazyme Biotech Co., Ltd (Nanjing, China) (Additional file 1: Figure S4). The gene encoding YsADH was introduced into the sites *Nco*I/*Hin*dIII of the vector pACYCDuet-1, yielding the recombinant plasmid pACYCDuet-1-*YsADH*. Moreover, the gene encoding TkNOX was further inserted into the sites *Nd*eI/*Xho*I of the plasmid pACYCDuet-1-*YsADH*, yielding the recombinant plasmid pACYCDuet-1-*YsADH*-*TkNOX*. In addition, the gene encoding VsHGB was introduced into the sites *Eco*RI and *Hin*dIII of the vector pET28a, offering the recombinant plasmid pET28a-*VsHGB*.

The recombinant plasmids pACYCDuet-1-*YsADH* and pACYCDuet-1-*YsADH*-*TkNOX* were transformed into the host strain *E. coli* BL21(DE3), giving rise to the recombiant strains *E. coli* BL21(DE3)/pACYCDuet-1-*YsADH* and *E. coli* BL21(DE3)/pACYCDuet-1-*YsADH*-*TkNOX*, respectively. Furthermore, the recombinant plasmid pET28a-*VsHGB* was transformed into the strain *E. coli* BL21(DE3)/pACYCDuet-1-*YsADH*-*TkNOX*, leading to the strain *E. coli* BL21(DE3)/pACYCDuet-1-*YsADH*-*TkNOX*/pET28a-*VsHGB.*

The recombinant *E. coli* strains were routinely grown in LB medium containing 50 μg/ml chloramphenicol and/or 50 μg/ml kanamycin at 37 °C until the OD_600_ of 0.6–0.8. Specifically, chloramphenicol was used for the cells containing the vector pACYCDuet-1, kanamycin was used for cells containing the vector pET28a, meanwhile both chloramphenicol and kanamycin were supplemented for cells containing both pACYCDuet-1 and pET28a vectors. Strains were induced by adding 0.3 mM IPTG and cultured at 20 °C for 12 h. Cells were washed twice using 50 mM Tris-HCl buffer (pH 8.0) and then harvested by 8000 *g* centrifugation at 4 °C for 10 min. Finally, lyophilized cells were obtained by freeze-drying and stored at − 20 °C for further use.

### Construction and overexpression of the YsADH-(linker)-TkNOX and YsADH-(linker)-TkNOX-(linker)-VsHGB fusion enzymes in *E. coli* cells

The fusion genes encoding YsADH, TkNOX and VsHGB were constructed by multiple overlap extension PCR [38]. To assembly four YsADH-(linker)-TkNOX fusion genes, the stop codon of the YsADH gene was removed, and the linkers with different lengths, (GSG)n (n = 1, 2) or (GGGGS)n (n = 1, 2), were introduced between the open reading frames of the YsADH and TkNOX genes via two rounds of PCR. The first round of PCR introduced the linkers (GSG)n (n = 1, 2) and (GGGGS)n (n = 1, 2) into the YsADH gene using four pairs of primers (Table 3). Simultaneously, the complementary linkers (GSG)n (n = 1, 2) and (GGGGS)n (n = 1, 2) were introduced into the TkNOX gene using four other pairs of primers (Table 3). Each PCR product was purified and served as a template in the second round of PCR. The PCR program included a 4 min period at 98 °C, 32 cycles at 98 °C (10 s), 58 °C (10 s) and 72 °C (30 s), and a final 5 min extension at 72 °C. The gel purified PCR products were ligated into a pACYCDuet-1 vector. Next, the PCR products of the YsADH and TkNOX genes were joined by overlapping extension PCR. The PCR program included a 4 min period at 98 °C, 32 cycles at 98 °C (10 s), 58 °C (10 s) and 72 °C (30 s), and a final 5 min extension at 72 °C. The gel purified PCR products were ligated into a pACYCDuet-1 vector. The four fusion genes were confirmed by sequencing. Finally, the four fusion genes were ligated to pACYCDuet-1 between *Nco*I and *Hin*dIII sites, yielding pACYCDuet-1-*YsADH-*(GGGGS)-TkNOX, pACYCDuet-1-*YsADH*-(GGGGS)_2_-*TkNOX*, pACYCDuet-1-*YsADH*-(GSG)-*TkNOX* and pACYCDuet-1-*YsADH*-(GSG)_2_-*TkNOX*. Using the construction of the plasmid pACYCDuet-1-*YsADH-*(GSG)-*TkNOX* as an example, the procedure was depicted in Additional file 1: Figure S5. Each expression construct pACYCDuet-1-*YsADH*-(GGGGS)-*TkNOX*, pACYCDuet-1-*YsADH*-(GGGGS)_2_-*TkNOX*, pACYCDuet-1-*YsADH*-(GSG)-*TkNOX* or pACYCDuet-1-*YsADH*-(GSG)_2_-*TkNOX*, was transformed into *E. coli* BL21(DE3) grown in LB medium containing 50 μg/ml chloramphenicol.

**Table 3:** The primers used for the construction of the fusion enzyme YsADH-(linker)-TkNOX, YsADH-(GSG)-TkNOX-(GSG)-VsHGB or VsHGB-(GSG)-TkNOX-(GSG)-YsADH

Linker	Gene		Primers
GGGGS	ADH	F	5′-TAACTTTAATAAGGAGATATACCATGGGCATGTCTATTATAAAAAGCTATGCC-3′
		R	5′-ACCACGGTTTTACGTTCCATGCTGCCGCCGCCGCCAAAGTCGGCTTGCAGTACCAC-3′
	TkNOX	F	5′-TGGTACTGCAAGCCGACTTTGGCGGCGGCGGCAGCATGGAACGTAAAACCGTGGTG-3′
		R	5′-ACTTAAGCATTATGCGGCCGCAAGCTTTCAAAATTTCAGAACACGTGCC-3′
GSG	ADH	F	5′-TAACTTTAATAAGGAGATATACCATGGGCATGTCTATTATAAAAAGCTATGCC-3′
		R	5′-CACCACGGTTTTACGTTCCATGCCGCTGCCAAAGTCGGCTTGCAGTACCAC-3′
	TkNOX	F	5′-GTGGTACTGCAAGCCGACTTTGGCAGCGGCATGGAACGTAAAACCGTGGTG-3′
		R	5′-ACTTAAGCATTATGCGGCCGCAAGCTTTCAAAATTTCAGAACACGTGCC-3′
(GGGGS)_2_	ADH	F	5′-TAACTTTAATAAGGAGATATACCATGGGCATGTCTATTATAAAAAGCTATGCC-3′
		R	5′-CACCACGGTTTTACGTTCCATGCTGCCGCCGCCGCCGCTGCCGCCGCCGCCAAAGTCGGCTTGCAGTACCAC-3′
	TkNOX	F	5′-GTGGTACTGCAAGCCGACTTTGGCGGCGGCGGCAGCGGCGGCGGCGGCAGCATGGAACGTAAAACCGTGGTG-3′
		R	5′-ACTTAAGCATTATGCGGCCGCAAGCTTTCAAAATTTCAGAACACGTGCC-3′
(GSG)_2_	ADH	F	5′-TAACTTTAATAAGGAGATATACCATGGGCATGTCTATTATAAAAAGCTATGCC-3′
		R	5′-CACCACGGTTTTACGTTCCATGCCGCTGCCGCCGCTGCCAAAGTCGGCTTGCAGTACCAC-3′
	TkNOX	F	5′-GTGGTACTGCAAGCCGACTTTGGCAGCGGCGGCAGCGGCATGGAACGTAAAACCGTGGTG-3′
		R	5′-ACTTAAGCATTATGCGGCCGCAAGCTTTCAAAATTTCAGAACACGTGCC-3′
GSG	YsADH-(GSG)-TkNOX	F	5′-TAACTTTAATAAGGAGATATACCATGGGCATGTCTATTATAAAAAGCTATGCC-3′
		R	5′-GGTCTGCTGGTCCAGCATGCCGCTGCCAAATTTCAGAACACGTGCC-3′
	VsHGB	F	5′-GGCACGTGTTCTGAAATTTGGCAGCGGCATGCTGGACCAGCAGACC-3′
		R	5′-ACTTAAGCATTATGCGGCCGCAAGCTTTTATTCAACTGCCTGAGCG-3′
GSG	VsHGB	F	5′-TAACTTTAATAAGGAGATATACCATGGATGCTGGACCAGCAGACC-3′
		R	5′-ATAACCACCACGGTTTTACGTTCCATGCCGCTGCCTTCAACTGCCTGAGCG-3′
	TkNOX	F	5-’ATCTGTACGCTCAGGCAGTTGAAGGCAGCGGCATGGAACGTAAAACCGTGG-3′
		R	5′-CGGCATAGCTTTTTATAATAGACATGCGCCGCTGCCAAATTTCAGAACACGTGCC-3′
	YsADH	F	5′-GGCACGTGTTCTGAAATTTGGCAGCGGCGCATGTCTATTATAAAAAGCTATGCCG-3′
		R	5′-ACTTAAGCATTATGCGGCCGC AAGCTTTCAAAAGTCGGCTTGCAG-3′

During the construction of YsADH-(linker)-TkNOX-(linker)-VsHGB fusion gene, the linker was chosen as GSG. Similar to the construction of YsADH-(linker)-TkNOX fusion genes, the fusion gene encoding YsADH-(GSG)-TkNOX-(GSG)-VsHGB or VsHGB-(GSG)-TkNOX-(GSG)-YsADH was obtained via two rounds of PCR and confirmed by sequencing. The fusion gene was ligated to pACYCDuet-1 between *Nco*I and *Hin*dIII sites and the resulting recombinant plasmid pACYCDuet-1-*YsADH*-(GSG)-*TkNOX*-(GSG)-*VsHGB* or pACYCDuet-1-*VsHGB*-(GSG)-*TkNOX*-(GSG)-*YsADH*. Using the construction of the plasmid pACYCDuet-1-*YsADH*-(GSG)-*TkNOX*-(GSG)-*VsHGB* as an example, the procedure was depicted in Additional file 1: Figure S6. Then, recombinant plasmid pACYCDuet-1-*YsADH*-(GSG)-*TkNOX*-(GSG)-*VsHGB* or pACYCDuet-1-*VsHGB*-(GSG)-*TkNOX*-(GSG)-*YsADH* was transformed into *E. coli* BL21(DE3) grown in LB medium containing 50 μg/ml chloramphenicol. Following the same procedure for the induction mentioned above, cells expressing the fusion enzyme YsADH-(linker)-TkNOX, YsADH-(GSG)-TkNOX-(GSG)-VsHGB or VsHGB-(GSG)-TkNOX-(GSG)-YsADH were induced, harvested and lyophilized.

### Enzyme assays

Lyophilized cells were re-suspended in the 50 mM Tris-HCl buffer (pH 8.0) and disrupted through ultrasonication for 10 min. After that, the cell-debris pellet and cell-free extract were separated by 17,000 *g* centrifugation at 4 °C for 10 min. Then, the cell-debris pellet was re-suspended to the same volume of cell-free extract using the 50 mM Tris-HCl buffer (pH 8.0). Finally, the cell-debris pellet and cell-free extract samples were run by SDS-PAGE (12% acrylamide in the resolving gel) and stained with Coomassie Brilliant Blue R-250 [39].

TkNOX activities in cell-free extracts were determined according to the previously-reported procedure [20]. YsADH activities in cell-free extracts were measured at 45 °C by monitoring the change of the absorbance at 340 nm. The enzyme assay for alcohol oxidation was carried out at 45 °C in triplicate in a reaction mixture (2.5 ml) composed of 20 mM crotyl alcohol and 1 mM NADP^+^ in 50 mM Tris-HCl (pH 8.0) buffer. The reaction was started by the addition of the enzyme. One unit of the activity is defined as formation or oxidation of 1 μmol NADPH per min. The protein concentrations of all samples were determined using the Bradford reagent with bovine serum albumin as the standard protein [40]. In addition, the determination of H_2_O_2_ was conducted according to the previously-reported procedure [33].

### The reaction mixture of α,β-unsaturated alcohol oxidation and its optimization

The setup of the reactor with hot plate/magnetic stirrer was shown in Additional file 1: Figure S7, in which the three-neck flask with magnetic stirring bar was used as a reaction vessel and the balloon was used to fill the atmosphere of reactor with oxygen. The standard reaction mixture (5 ml) contained 100 mM α,β-unsaturated alcohols, 0.3 g lyophilized cells, 0.4 mM NADP^+^, 0.2 mM FAD, 50 mM Tris-HCl buffer (pH 8.0). The reaction was carried out at 45 °C and 600 rpm for 2 h. The optimal temperature of alcohol oxidation was determined at a series of temperatures ranging from 40 to 65 °C. The optimal pH was determined over a range of pH 6.0 to 9.5 at 45 °C. The buffers used were PIPES (pH 6.0, 6.5 and 7.0), Tris-HCl (pH 7.5, 8.0, 8.5 and 9.0), and CAPS (pH 9.5). The optimal rotation was determined over a range of 400 to 900 rpm. FAD and NADP^+^ concentrations were explored within the range of 0 to 1 mM. After alcohol oxidation, the reaction mixture was extracted with 5 ml of ethyl acetate under strong vibration. The organic phase in samples was separated by 8000 *g* centrifugation at room temperature for 10 min and dehydrated by anhydrous sodium sulfate, and then 1 μl dehydrated sample was applied for GC analysis.

### Determination of substrates, products and possible by-products by gas chromatograph

α,β-Unsaturated alcohols/aldehydes and possible by-products were determined by GC (Agilent 6890N) equipped with an FID detector and chiral capillary BGB-174 column (BGB Analytik, Böckten, Switzerland, 30 m × 250 µm × 0.25 µm). The flow rate and split ratio of N_2_ as the carrier gas were set as 1.38 ml/min and 1:100, respectively. Both injector and detector were kept at 250 °C. The injection volume was 1 µl.

For crotyl alcohol/crotonaldehyde, 3-methyl-2-buten-1-ol/3-methyl-2-butenal, trans-2-hexenol/trans-2-hexenal and cinnamyl alcohol/cinnamaldehyde, the column temperature program was listed as follows: initial temperature of 75 °C for 3 min, 10 °C/min ramp to 120 °C for 3 min, and 30 °C/min ramp to 180 °C for 3 min. For geraniol/geranal and nerol/neral, the column temperature program was listed as follows: initial temperature of 75 ° for 3 min, 4 °C/min ramp to 120 °C for 3 min, and 30 °C/min ramp to 180 °C for 3 min. The retention times of the above-mentioned substrates and products were summarized in Table 4.

**Table 4:** The retention times of substrates and products in GC analysis

Entry	Alcohols	Time (min)	Aldehydes	Time (min)
1	Crotyl alcohol	3.509	Crotonaldehyde	3.777
2	3-Methyl-2-buten-1-ol	5.320	3-Methyl-2-butenal	7.752
3	*Trans*-2-Hexenol	6.115	*Trans*-2-Hexenal	13.151
4	Nerol	17.619	Neral	18.858
5	Geraniol	18.562	Geranial	19.504
6	Cinnamyl alcohol	14.851	Cinnamaldehyde	14.117
7	Farnesol	19.662	Farnesal	19.471

For possible by-products isovaleraldehyde, isovaleric acid and 3,3-dimethylacrylic acid, the column temperature program was listed as follows: initial temperature of 60 °C for 5 min, 10 °C/min ramp to 120 °C for 3 min, and 30 °/min ramp to 180 °C for 3 min. The retention times for isovaleraldehyde, 3,3-dimethylacrylic acid and isovaleric acid were 6.987 min, 7.839 min and 9.612 min, respectively.

### HPLC-based determination of retinol and retinal

Retinol and retinal were determined by HPLC (Waters 2010) equipped with an UV detector and C-18 column (Welch, 30 m × 250 µm × 0.25 µm). The HPLC conditions were listed as follows: Temperature, 40 °C; the wavelength of UV detector, 340 nm; mobile phase, methanol:acetonitrile = 95:5; flow rate, 1 ml/min. The retention times for retinol and retinal were 5.237 min and 5.618 min, respectively.

## Supplementary Information

**Additional file 1: Table S1.** The activities for YsADH and TkNOX in the cell-free extracts of whole-cell catalysts. **Figure S1.** SDS-PAGE analysis of cell-free extract and cell-debris pellet from the same whole catalyst comprising YsADH, TkNOX and/or VsHGB. **Figure S2.** SDS-PAGE analysis of cell-free extract and cell-debris pellet from the same whole catalyst comprising the fusion enzyme of YsADH and TkNOX. **Figure S3.** SDS-PAGE analysis of cell-free extract and cell-debris pellet from the same whole catalyst comprising the fusion enzyme of YsADH, TkNOX and VsHGB. **Figure S4.** The codon-optimized nucleotide sequences encoding YsADH (a), TkNOX (b) and VsHGB (c). **Figure S5.** The construction of the plasmid pACYCDuet-1-*YsADH*-(GSG)-*TkNOX.* **Figure S6.** The construction of the plasmid pACYCDuet-1-*YsADH*-(GSG)-*TkNOX*-(GSG)-*VsHGB.* **Figure S7.** The reactor with hot plate/magnetic stirrer (a) and its key components (b).

## Data Availability

The gene sequences in this study are available in the GenBank with Accession number of KF887947 for YsADH, BAD85488 for TkNOX and AAA27584 for VsHGB.

## Abbreviations

YsADH: Alcohol dehydrogenase from *Yokonella* sp. WZY002
TkNOX: NADPH oxidase from *Thermococcus kodakaraensis*
VsHGB: Hemoglobin from *Vitreoscilla stercoraria*
FAD: Flavin adenine dinucleotide
NADP: Nicotinamide adenine dinucleotide phosphate
SDS-PAGE: Sodium dodecyl sulfate polyacrylamide gel electrophoresis
IPTG: Isopropyl β-d-1-thiogalactopyranoside
LB: Luria–Bertani
GC: Gas chromatography
FID: Flame ionization detector
HPLC: High performance liquid chromatography
PIPES: Piperazine-1,4-bisethanesulfonic acid
CAPS: *N*-Cyclohexyl-3-aminopropanesulfonic acid
