# Supplementary material for: Efficient whole-cell oxidation of α,β-unsaturated alcohols to α,β-unsaturated aldehydes through the cascade biocatalysis of alcohol dehydrogenase, NADPH oxidase and hemoglobin
Source: Microb Cell Fact. 2021 Jan 19;20:17. doi: 10.1186/s12934-021-01511-8 (PMC7816460; doi:10.1186/s12934-021-01511-8)
Supplement: Supplementary file 1 — Additional file 1: Table S1. The activities for YsADH and TkNOX in the cell-free extracts of whole-cell catalysts. Figure S1. SDS-PAGE analysis of cell-free extract and cell-debris pellet from the same whole catalyst comprising YsADH, TkNOX and/or VsHGB. Figure S2. SDS-PAGE analysis of cell-free extract and cell-debris pellet from the same whole catalyst comprising the fusion enzyme of YsADH and TkNOX. Figure S3. SDS-PAGE analysis of cell-free extract and cell-debris pellet from the same whole catalyst comprising the fusion enzyme of YsADH, TkNOX and VsHGB. Figure S4. The codon-optimized nucleotide sequences encoding YsADH (a), TkNOX (b) and VsHGB (c). Figure S5. The construction of the plasmid pACYCDuet-1-YsADH-(GSG)-TkNOX. Figure S6. The construction of the plasmid pACYCDuet-1-YsADH-(GSG)-TkNOX-(GSG)-VsHGB. Figure S7. The reactor with hot plate/magnetic stirrer (a) and its key components (b). [file 12934_2021_1511_MOESM1_ESM.docx]

Additional Information

Efficient whole-cell oxidation of α,β-unsaturated alcohols to α,β-unsaturated aldehydes through the cascade biocatalysis of alcohol dehydrogenase, NADPH oxidase and hemoglobin

Yan Qiao^1^, Can Wang^1^, Yin Zeng^1^, Tairan Wang^1^, Jingjing Qiao^1^, Chenze Lu^2^, Zhao Wang^1^ and Xiangxian Ying^1*^

^1^ Key Laboratory of Bioorganic Synthesis of Zhejiang Province, College of Biotechnology and Bioengineering, Zhejiang University of Technology, Hangzhou 310014, China;

^2^ College of Life Sciences, China Jiliang University, Hangzhou 310018, China;

* Correspondence: [yingxx@zjut.edu.cn](mailto:yingxx@zjut.edu.cn)

**Contents**

[Table S1. The activities for YsADH and TkNOX in the cell-free extract of whole-cell catalyst](#_Toc12339) 2

[Figure S1. SDS-PAGE analysis of cell-free extract and cell-debris pellet from the same whole catalyst comprising YsADH, TkNOX and/or VsHGB](#_Toc6085) 3

[Figure S2. SDS-PAGE analysis of cell-free extract and cell-debris pellet from the same whole catalyst comprising the fusion enzyme of YsADH and TkNOX](#_Toc18733) 4

[Figure S3. SDS-PAGE analysis of cell-free extract and cell-debris pellet from the same whole catalyst comprising the fusion enzyme of YsADH, TkNOX and VsHGB](#_Toc31881) 5

[Figure S4. The codon-optimized nucleotide sequences encoding YsADH (a), TkNOX (b) and VsHGB (c).](#_Toc31030) 6

[Figure S5. The construction of the plasmid pACYCDuet-1-](#_Toc30819)*[YsADH](#_Toc30819)*[-(GSG)-](#_Toc30819)*[TkNOX](#_Toc30819)*[.](#_Toc30819) 7

[Figure S6. The construction of the plasmid pACYCDuet-1-](#_Toc16967)*[YsADH](#_Toc16967)*[-(GSG)-](#_Toc16967)*[TkNOX](#_Toc16967)*[-(GSG)-](#_Toc16967)*[VsHGB](#_Toc16967)*[.](#_Toc16967) 8

[Figure S7. The reactor with hot plate/magnetic stirrer (a) and its key components (b)](#_Toc29499) 9

Additional tables

# Table S1. The activities for YsADH and TkNOX in the cell-free extract of whole-cell catalyst

| **The enzyme(s) expressed in the whole cell catalyst** | **YsADH activity (U/g)** | **TkNOX activity (U/g)** |
| --- | --- | --- |
| YsADH | 3568 ± 102 |  |
| YsADH, TkNOX | 650 ± 22 | 1618 ± 46 |
| YsADH, TkNOX and VsHGB | 613 ± 24 | 1542 ± 35 |
| YsADH-(GSG)-TkNOX | 797 ± 31 | 1156 ± 51 |
| YsADH-(GSG)_2_-TkNOX | 783 ± 30 | 1128 ± 37 |
| YsADH-(GGGGS)-TkNOX | 780 ± 19 | 1154 ± 26 |
| YsADH-(GGGGS)_2_-TkNOX | 775 ± 16 | 1087 ± 30 |
| YsADH-(GSG)-TkNOX-(GSG)-VsHGB | 801 ± 28 | 983 ± 23 |
| VsHGB-(GSG)-TkNOX-(GSG)-YsADH | 752 ± 17 | 1064 ± 33 |

Additional figures


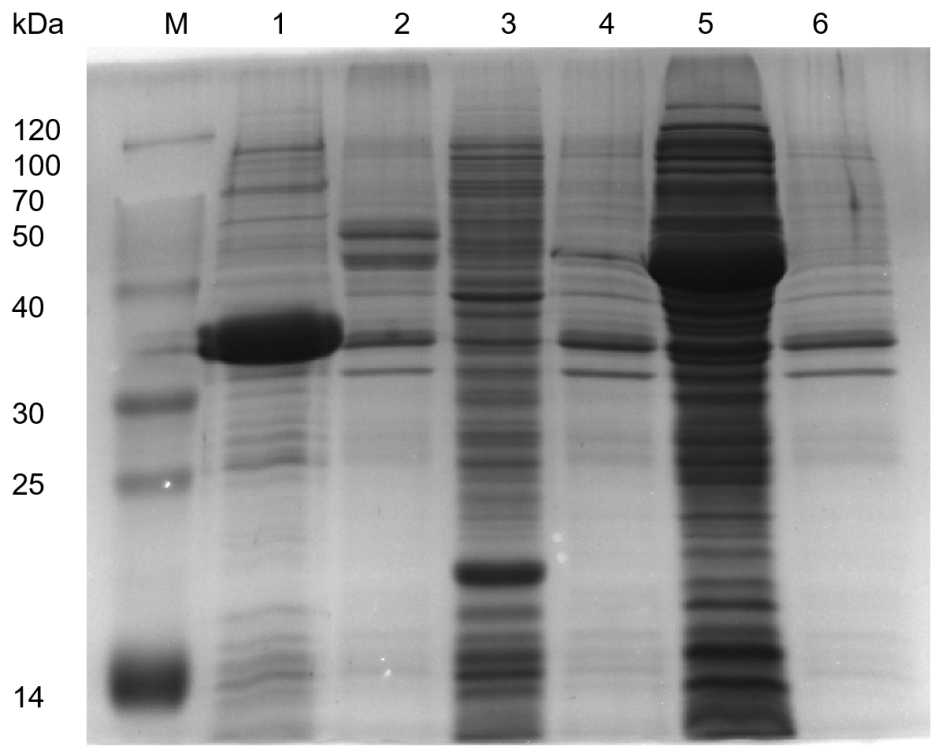


Figure S1. SDS-PAGE analysis of cell-free extract and cell-debris pellet from the same whole catalyst comprising YsADH, TkNOX and/or VsHGB. Lane M, marker; lane 1 and 2, cell-free extract and cell-debris pellet from the catalyst comprising YsADH (34 kDa); lane 3 and 4, cell-free extract and cell-debris pellet from the catalyst comprising VsHGB (18 kDa); lane 5 and 6, cell-free extract and cell-debris pellet from the catalyst comprising YsADH and TkNOX (44 kDa). The percentage of acrylamide in the resolving gel was 12%. The value in the bracket represents the apparent molecular mass of YsADH, TkNOX or VsHGB.


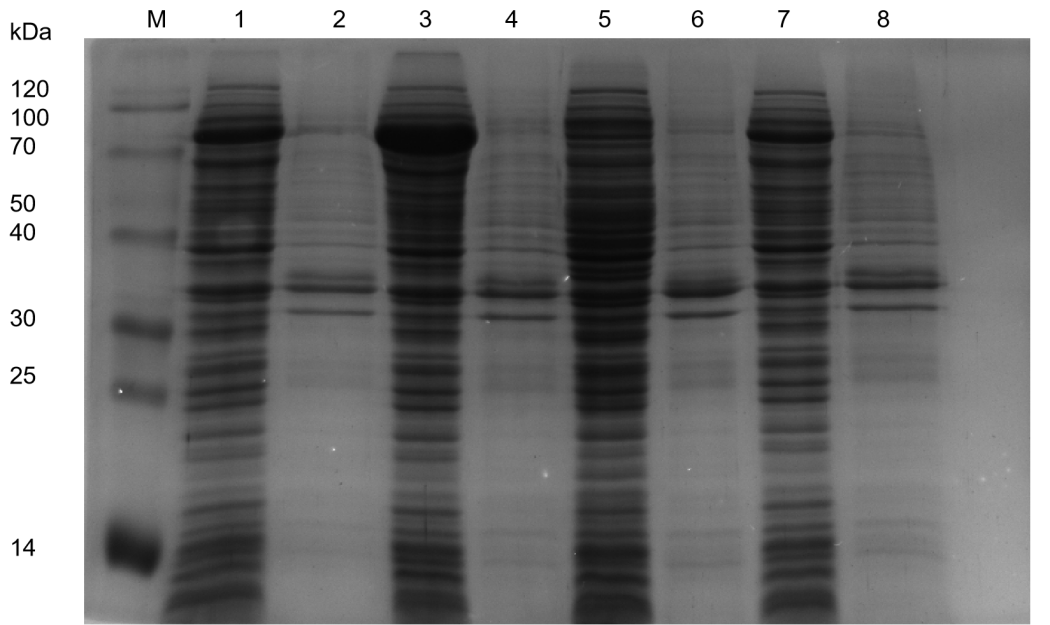


Figure S2. SDS-PAGE analysis of cell-free extract and cell-debris pellet from the same whole catalyst comprising the fusion enzyme of YsADH and TkNOX (80 kDa). Lane M, marker; lane 1 and 2, cell-free extract and cell-debris pellet from the catalyst comprising YsADH-(GGGGS)-TkNOX; lane 3 and 4, cell-free extract and cell-debris pellet from the catalyst comprising YsADH-(GSG)-TkNOX; lane 5 and 6, cell-free extract and cell-debris pellet from the catalyst comprising YsADH-(GGGGS)_2_-TkNOX; lane 7 and 8, cell-free extract and cell pellet from the catalyst comprising YsADH-(GSG)_2_-TkNOX. The percentage of acrylamide in the resolving gel was 12%. The value in the bracket represents the apparent molecular mass of the fusion enzyme.


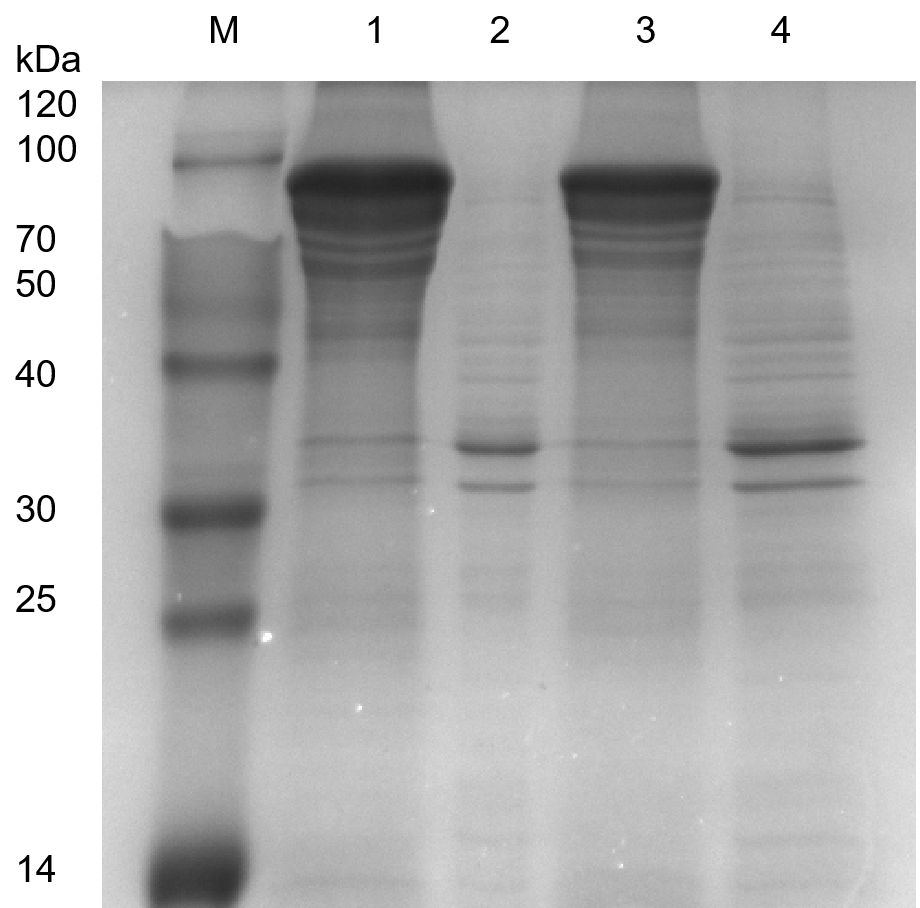


Figure S3. SDS-PAGE analysis of cell-free extract and cell-debris pellet from the same whole catalyst comprising the fusion enzyme of YsADH, TkNOX and VsHGB (95 kDa). Lane M, marker; lane 1 and 2, cell-free extract and cell-debris pellet from the catalyst comprising YsADH-(GSG)-TkNOX-(GSG)-VsHGB; lane 3 and 4, cell-free extract and cell-debris pellet from the catalyst comprising VsHGB-(GSG)-TkNOX-(GSG)-YsADH. The percentage of acrylamide in the resolving gel was 12%. The value in the bracket represents the apparent molecular mass of the fusion enzyme.

atgtctattataaaaagctatgccgcaaaagaggcgggcagcgaactcgaactttacgaatatgatgccggtgaactcaggccggaagatgtcgaggtgcaggtcgactactgcggtatctgccattccgatctttccatgatcgacaacgaatggggattctctcagtatccgctggttgccgggcatgaagtgattggccgcgtggcggcgctcggcagtgcggcgcaggaaaaaggggtgaaagttggtcagcgcgtgggcgtaggctggacggcgcgcagctgtgggcattgcgatgcatgtatcagcggtaatcagattaactgcctggaaggcgccgtagccaccattctcaaccgtggcggttttgccgagaaactgcgggcagactggcagtgggtgatcccgcttccggagagcatcgatattgagtcggcaggtcctctgttatgcggcggtattacggtttttaaacctctgctgatgcaccacatcaccgcgaccagtcgcgtgggggtgatcggcatcggcggtcttgggcacattgccattaaactgttgcacgcaatgggctgtgaagtgaccgcattcagctcgaatccgtcgaaagaacaggaagtgctggcaatgggggcggataaagtcgtgaacagtcgcgatccagacgcgttaaatgcgctggcaggccagtttgatctcattatcaacaccgttaatgtcgacctcgactggcagccctactttgaagcgctggcctatggcggccatttccacaccgtcggcgcagtgatgaagccgctgccggttccggcgtttacattgattgctggcgatcgcagcatctccggctcagcaaccggtacgccctatgagctgcgcaaattgatgaagtttgccgggcgcagcaaggtctcgccgacgacagagctgttcccaatgtcgcaaatcaacgaagccatccagcacgttcgcgacggcaaagcgcgttaccgcgtggtactgcaagccgacttttga

（a）

atggaacgtaaaaccgtggtggttattggtggtggtgcggccggtatgagcaccgcgagccgtgtgaaacgtctgaaaccggaatgggatgtgaaagtttttgaagcaaccgaatgggtgagccatgcaccgtgtggaattccgtatgttgttgagggaattagcccgaaagaaaaactgatgcattatccgccggaagtgtttattaaaaagcgtggtattgatctgcatctgaaagcagaagttattgaggttgaacagggtcgtgttcgtgtgcgtgaagaagatggagaaaaaacctatgaatgggattatctggtttttgcaaatggtgcaagcccgcaggttccggcaattgaaggtattgatctgccgggtgtttttaccgcagatctgccgccggatgcagttgcaattaccgaatatctggaaaaaaatccggttgaaaatgttgttgttattggtaccggttatattgcaattgaaatggcagaagcatttgttgaacgtggtaaaaatgttaccctgattggtcgtagcgaacgtgttctgcgtaaaacctttgataaagaaattaccgatattgttgaagaaaaactgcgtaatcatctgaatctgcgtctggaagaagttaccctgcgtattgaaggtaaagaacgtgttgaacgtgttgttaccgatgcaggtgaatatccggcagatctggttattgttgcaaccggtattaaaccgaataccgaactggcacgtggtctgggtgttcgtattggtgaaaccggtgcaatttggaccaatgatcgtatgcagaccagcgttgaaaatgtttatgcagcaggtgatgttgcagaaaccaaacatctgattaccggtcgtcgtgtttggatgccgctggcaccggcaggtaataaaatgggttatgttgcaggtagcaatattgcaggtaaagaaattcattttccgggtgttctgggtaccagcattaccaaatttctggatctggaaattggtaaaaccggtctgaccgaagcagaagcaatgaaagaaggttatgatgttcgtaccgcatttattaaagcaggtacccgtccgcattattatccgggtagcaaaaccatttggctgaaaggtgttgttgataatgaaaccaatcgtctgctgggtgttcaggcagttggtggtgatattctgccgcgtattgataccgcagcagcaatgattaccgcaggttttaccaccaaagatgttttttttaccgatctggcatacgctccgccgtttgcaccggtttgggatccgctgattgttctggcacgtgttctgaaattt

(b)

atgctggaccagcagaccattaatattat1taaagccaccgttccggtgctgaaagaacacggggtgaccattaccaccaccttttataaaaacctgtttgccaaacacccggaagttcgccctctgtttgatatgggtcgccaggagagcctggaacagccaaaagcactggcaatgaccgttctggcagcagcacagaatatcgaaaacctgcctgcaatcctgcctgcagtgaaaaagattgccgtgaaacattgtcaggcaggagtcgcagcagcacactatcctattgtgggccaagaactgctgggtgcaatcaaagaagtcctgggtgatgcagcaacagatgatattctggacgcatggggtaaagcctatggagtgattgcagatgtttttattcaggtggaagcagatctgtacgctcaggcagttgaataa

(c)

Figure S4. The codon-optimized nucleotide sequences encoding YsADH (a), TkNOX (b) and VsHGB (c).

**
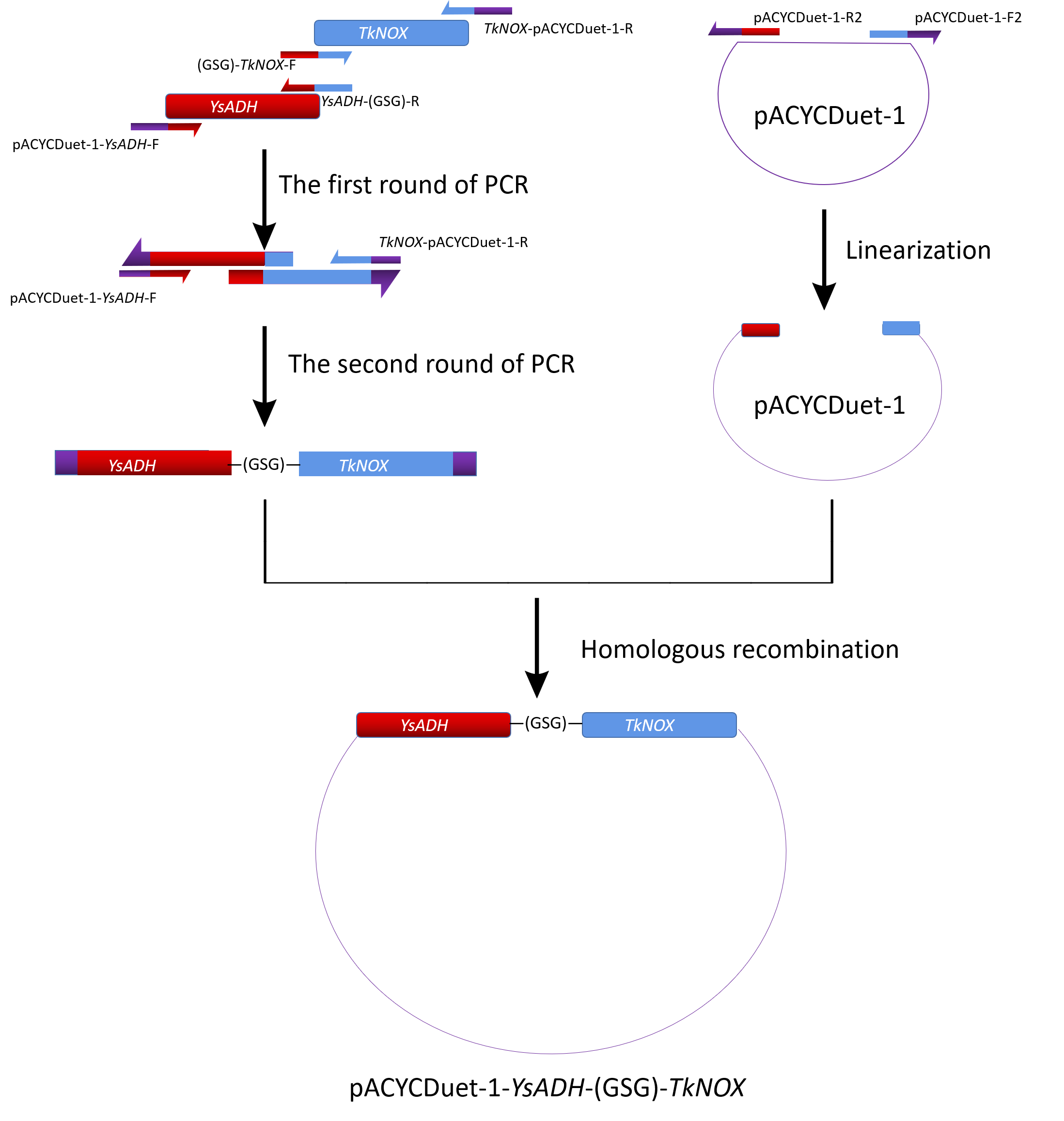
**

Figure S5. The construction of the plasmid pACYCDuet-1-*YsADH*-(GSG)-*TkNOX*. The primer pACYCDuet-1-R2：5’-TGCGGCCGCATAAATGTCTA-3’; pACYCDuet-1-F2：5’-TATATCTCCTTATTAAAGTTAAACAAAAAATTTC-3’. Other primers were summarized in Table 3.

#

#
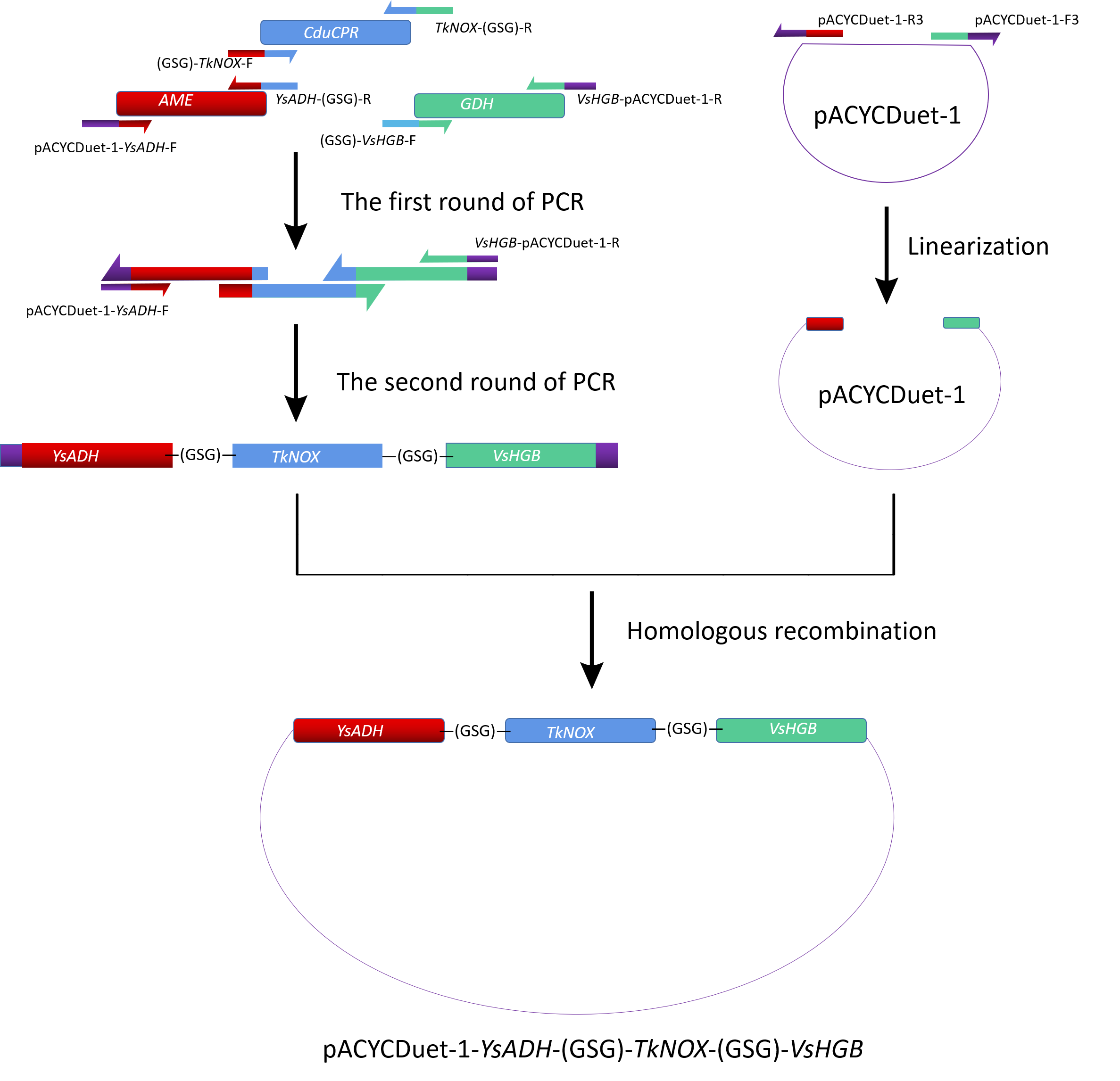


# Figure S6. The construction of the plasmid pACYCDuet-1-*YsADH*-(GSG)-*TkNOX*-(GSG)-*VsHGB*. The primer pACYCDuet-1-R3：5’-TGCGGCCGCATAAATGTCTA-3’; pACYCDuet-1-F3：5’-ATATCTCCTTATTAAAGTTAAACAAATTATTCAAC-3’. Other primers were summarized in Table 3.


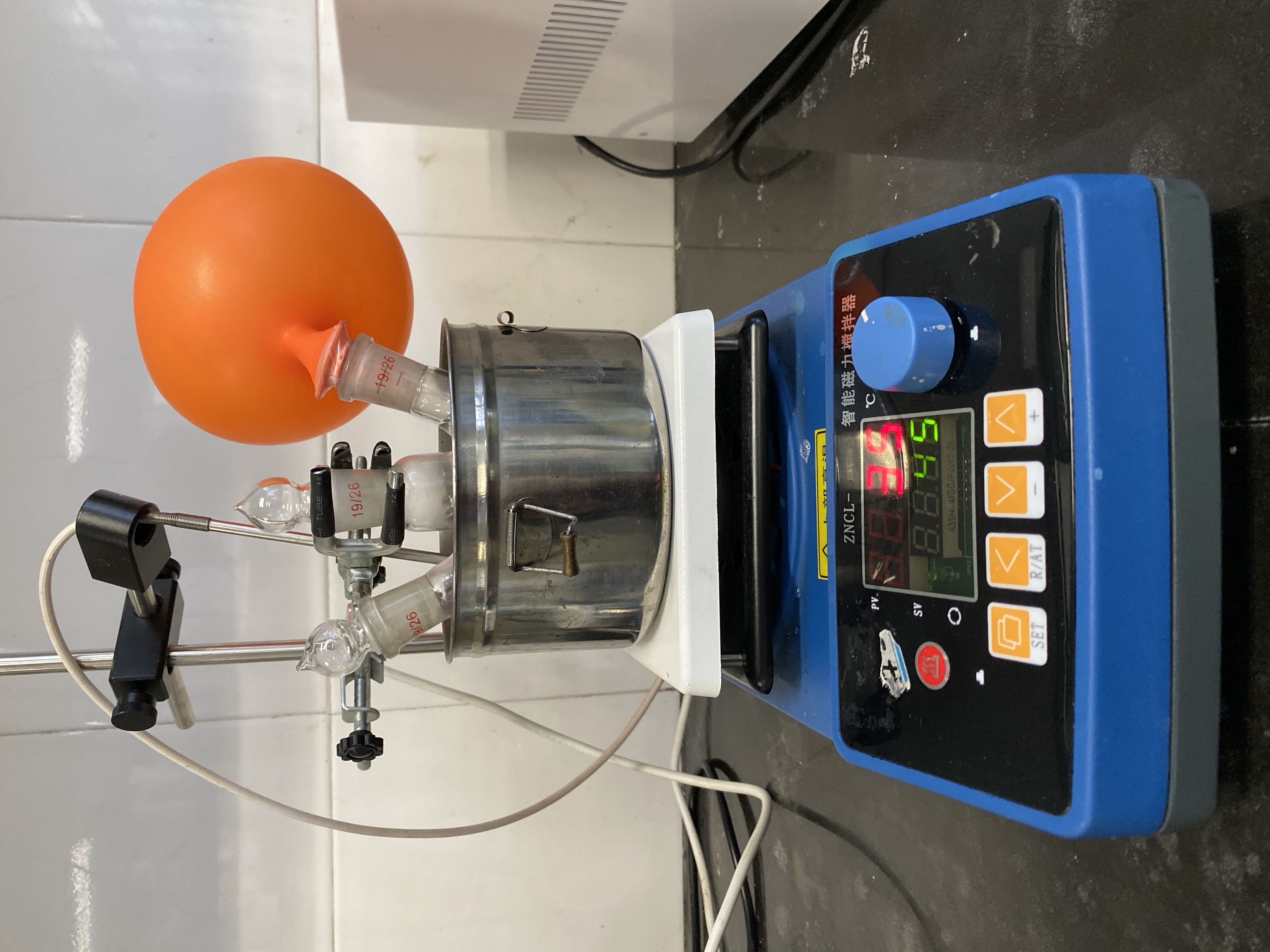

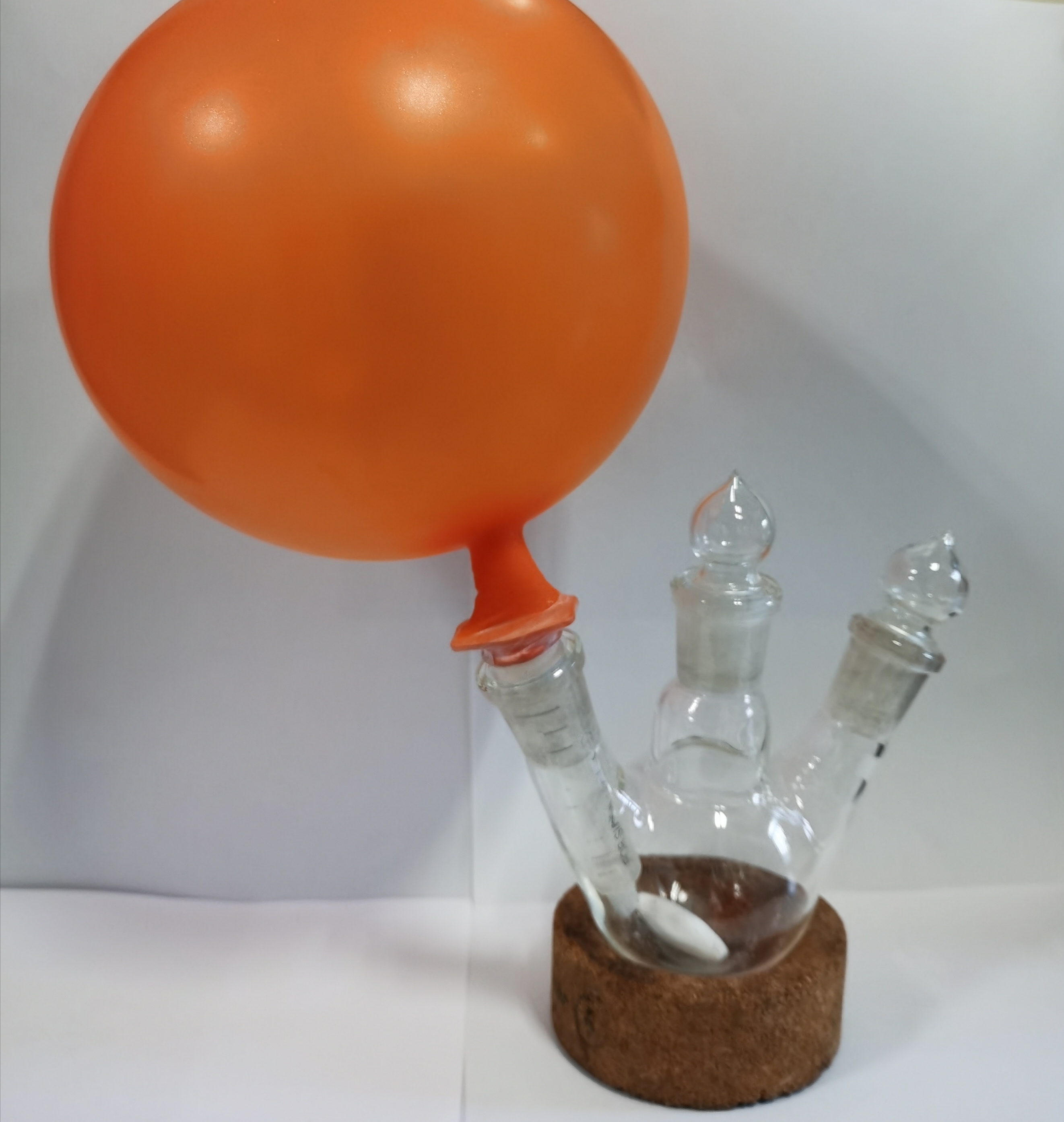


1. (b)

Figure S7. The reactor with hot plate/magnetic stirrer (a) and its key components (b). The three-neck flask with magnetic stirring bar was used as a reaction vessel and the balloon was used to fill the atmosphere of reactor with oxygen.
